# Echoes from the past: adjustment of aging former prisoners of war to the COVID-19 pandemic

**DOI:** 10.1017/S003329172100115X

**Published:** 2021-03-18

**Authors:** Karni Ginzburg, Mario Mikulincer, Avi Ohry, Zahava Solomon

**Affiliations:** 1 The Bob Shapell School of Social Work, Tel Aviv University, Israel; 2 School of Psychology, Interdisciplinary Center (IDC) Herzliya, Israel; 3 Sackler Faculty of Medicine, Tel Aviv University and the Reuth Medical and Rehabilitation Center, Tel Aviv, Israel

**Keywords:** COVID-19, acute stress disorder, prisoners of war

## Abstract

**Background:**

The aim of this study was to examine whether exposure to previous traumatic events is a risk factor for stress reactions during this pandemic. Capitalizing on a 29-year longitudinal study of Israeli ex-prisoners of war (ex-POWs) and combat veterans, we examined whether captivity is a risk factor for fear of coronavirus disease 2019 (COVID-19) and COVID-19-induced acute stress disorder (COVID-19 ASD) beyond the effects of combat exposure and other stressful life events. In addition, we examined the contribution of captivity experiences (severity of captivity, experience of solitary confinement, and suffering during captivity) and veterans' appraisal of the impact of their war-related experiences on adjustment to the current quarantine and isolation to fear of COVID-19 and COVID-19 ASD.

**Methods:**

One-hundred-and-twenty Israeli ex-POWs from 1973 Yom Kippur War and 65 matched controls (combat veterans from the same war) filled out self-report questionnaires 18 (T1), 35 (T2), 42 (T3), and 47 (T4) years after the war.

**Results:**

Findings revealed that although ex-POWs and controls did not differ in their level of exposure to COVID-19, ex-POWS reported higher levels of fear of COVID-19 and COVID-19 ASD than controls. Suffering during captivity, measured at 1991, and participants' appraisal of the extent to which their war-related experiences affected adjustment to COVID-19 were significantly associated with fear of COVID-19 and COVID-19 ASD.

**Conclusions:**

The findings of the study demonstrate the long-term effects of exposure to traumatic experiences (captivity) during young adulthood on adjustment to an unrelated collective stress, such as COVID-19, 40 years later.

The coronavirus disease 2019 (COVID-19) pandemic is an intense global crisis. In addition to the threat of death, especially for older adults, it involves various stressors, including exposure to death, financial insecurity, exposure to discordant and mixed messages of health officials and political leaders, uncertainty regarding the future, forced quarantine, and social isolation. Studies have demonstrated elevated levels of fear of COVID-19 among the general population. Large-scale surveys conducted in China during the outbreak of the pandemic report high rates of COVID-19-induced acute stress disorder (COVID-19 ASD), ranging between 16% and 24% (Lin et al., 2021; Shi et al., 2020).

Although the pandemic seems to be highly stressful for a large proportion of the population, there are indirect indications that individuals who have been exposed to previous traumatic events may be at a particularly high risk for stress reactions. Studies show that traumatic life events during childhood predicted combat-related posttraumatic stress disorder (PTSD) among war veterans (Solomon, Zur-Noah, Horesh, Zerach, & Keinan, 2008). The pathogenic effect of prior life events was demonstrated among survivors of other traumatic events (Ginzburg, 2006).

At the same time, there are indications that it is not only the exposure to previous traumatic events but the way that survivors subjectively *link* their previous traumatic experiences to the current stressor that affects their adjustment. A study conducted among aging Israeli residents during the Persian Gulf War, during which the population was exposed to missile attacks and the threat of biological and chemical weapons, found that Holocaust survivors demonstrated only marginal higher levels of distress compared to that demonstrated by controls (Solomon & Prager, 1992). Among Holocaust survivors, however, those who reported that they had been previously exposed to events similar to the Gulf War reported higher levels of distress than those who had not (Hantman, Solomon, & Prager, 1994). Similarly, a study conducted among aging Holocaust survivors with cancer found that their perceptions regarding the extent to which their Holocaust experience made it difficult for them to handle the problems of aging were associated with their level of distress (Hantman & Solomon, 2007).

## The current study: effects of war captivity on adjustment to the COVID-19 pandemic

This study was conducted between April 12, 2020 and May 17, 2020. During the initial stage of data collection, there were 11 599 verified cases of COVID-19 in Israel, and 16 640 verified cases by the end of data collection. As for deaths related to COVID-19, there were 103 deaths in the initial stage and 271 cases by the end of data collection. During this time, the Israeli government had imposed a quarantine on the whole population. The entire population was under lockdown, with only essential and emergency services remaining open.

The aim of the current study was to examine fear of COVID-19 and COVID-19 ASD symptoms among Israeli aging ex-prisoners of war (ex-POWs). These individuals seem to be particularly vulnerable to the effects of the current crisis, not only because of their age, which puts them in a high-risk group, but also due to the nature of their traumatic experiences of captivity which, along with intimidation, torture, humiliation, and the threat of death, often involved confinement, movement restriction, and isolation. Many of them said that the most stressful element for them in captivity was social distancing, seclusion, and isolation in solitary confinement. Not a few reported that social isolation was so painful and distressing that they actually wished to be interrogated; despite the fact that interrogation was often extremely harsh, and involved the most painful physical torture, at least it involved interpersonal interaction (Stein, Snir, & Solomon, 2015). It is then reasonable to suggest that the COVID-19-imposed quarantine and directives for social isolation may have symbolically resembled their captivity experiences. Thus, the aim of this study was to examine the specific implications of war captivity experiences, beyond other stressful life experiences, for adjustment to COVID-19.

Capitalizing on a 29-year longitudinal study of Israeli ex-POWs and combat veterans of the 1973 Yom Kippur War who were evaluated at three previous time points – 1991 (T1), 2008 (T2), and 2015 (T3) – and then during the COVID-19 outbreak during April−May 2020 (T4), we examined the following research questions: (1) Is captivity a risk factor for fear of COVID-19 and COVID-19 ASD, beyond the effects of combat exposure and other stressful life events? (2) Do captivity experiences (the severity of captivity, experience of solitary confinement, and suffering during captivity), as measured at T1, predict fear of COVID-19 and COVID-19 ASD at T4? and (3) Is veterans' appraisal of the effect of their war-related experiences on adjustment to the current quarantine and isolation associated with fear of COVID-19 and COVID-19 ASD?

## Method

### Participants and procedure

Two hundred and forty Israeli ground forces soldiers were captured during the 1973 Yom Kippur War. In this study, 164 of these ex-POWs participated at T1, 183 at T2 (29 could not be located/refused, 20 had died, and six could not participate due to mental deterioration), and 158 at T3 (49 could not be located/refused, 30 had died, and three suffered from physical or mental problems). One hundred and twenty of these ex-POWs participated in the assessment conducted during the COVID-19 outbreak (T4, 66 could not be located/refused, 36 had died, and 18 could not participate due to mental deterioration).

In addition, 280 veterans were sampled from the Israel Defense Forces (IDF) computerized database (Solomon, Neria, Ohry, Waysman, & Ginzburg, 1994). These individuals also participated in the Yom Kippur War on the same fronts, but were not taken captive, and were matched to ex-POWs on military background and socio-demographic variables. Among them, 185 participated at T1, 118 took part at T2 (20 could not be located/refused, and five had died), and 101 participated at T3 (34 declined to participate, 14 could not be located, 2 did not return the questionnaire, 1 was abroad, and 18 had died). At T4, the target group included 136 controls; of those, 65 participated at the study (65 could not be located/refused, 3 had died, and 3 could not participate due to mental deterioration).

Data in the current study were anchored to include veterans who participated at the T4 measurement (see Newman, 2014) and had no missing data on fear of COVID-19 and COVID-19 ASD scales (*N* = 185, 120 ex-POWs and 65 controls). Participants' age, education, occupational status, and living status at T4 in the ex-POW and control groups are presented in Table 1. As can be seen, the two groups differed in age and occupational and living status. More specifically, ex-POWs were somewhat older than controls. More ex-POWs reported living with their spouses than the controls. Finally, more ex-POWs reported not working at T4, unrelated to the COVID-19 pandemic, as compared to controls. The groups did not differ in level of education.

**Table 1.** Differences between ex-POWs and controls in demographic and study variables

	Ex-POWs *N* = 120	Controls *N* = 65		
	*M* (*s.d.*)	*M* (*s.d.*)	*t*	*p*
Age (T4)	69.38 (4.33)	68.17 (2.84)	2.03	0.044
Education (T4)	14.57 (4.03)	14.75 (3.00)	0.31	0.761
Life events (T1)	6.20 (5.00)	5.75 (4.98)	0.59	0.56
Life events (T3)	3.45 (1.87)	3.28 (1.35)	0.66	0.47
COVID-19 exposure (T4)	1.26 (1.47)	1.14 (1.27)	0.55	0.58
Fear of COVID-19 (T4)	4.89 (0.86)	4.41 (0.93)	3.50	0.001
COVID-19 ASD (T4)	2.07 (0.84)	1.45 (0.54)	3.42	0.001
	*N* (*%*)	*N* (*%*)	χ^2^	*p*
Living with spouse (T4)	115 (95.8%)	56 (85.7%)	6.51	0.04
COVID-19-related occupational status (T4)			16.51	0.001
Working, no change	18 (15%)	25 (38.8%)		
Working, some change	22 (18.3%)	12 (18.5%)		
Not working due to COVID-19	18 (15%)	11 (16.9%)		
Not working, no change	62 (51.7%)	17 (26.2%)		
Appraisal of war experiences (T4)			25.02	<0.001
Facilitate	24 (20.0%)	7 (10.8%)		
No effect	51 (42.5%)	52 (80.0%)		
Hinder	45 (37.5%)	6 (9.2%)		

The appraisal of war experiences refers to the effect of war experiences on adjustment to COVID-19-induced lockdown and home isolation; life events at T3 represent the sum of number of life events at T2 and T3.

Data on captivity experience were collected at T1 (1991). Exposure to stressful life events was assessed at T1, T2, and T3. Data concerning level of exposure to COVID-19, appraisals of the extent to which their war-related experiences affected their current adjustment, fear of COVID-19, and COVID-19 ASD were assessed at T4. The study was approved by the institutional review board (IRB) and all participants signed a consent form.

### Measures

#### Background variables

At T4, participants were asked about their age, education, occupational status, and with whom they lived. At T1 and T2 participants completed a brief scale on *exposure to stressful life events*, which was designed by investigators familiar with life in Israel and used in studies of Israeli war veterans (Horesh, Cohen-Zrihen, Dor, & Solomon, 2014) and civilians (Ginzburg, 2006). Participants were asked to report whether or not they experienced a targeted event (e.g. death of a significant other, motor vehicle accident, severe illness, being a witness or a victim of a criminal act). At T3, participants were asked to list events they experienced since T2. For each participant, we computed the number of reported stressful events at each wave of measurement.

*Exposure to COVID-1*9 was assessed by 10 COVID-19-related incidents (e.g. becoming infected, having to go into quarantine, knowing someone who died from COVID-19) (Tsur & Abu-Raiya, 2020; Zhen & Zhou, 2020). Overall exposure was calculated by summing all positive answers to exposure questions, with higher scores indicating higher exposure to COVID-19. At T4, participants were also asked for their appraisals of whether their wartime experiences affected the way they adjusted to the current lockdown and social restrictions (1 = it facilitated their adjustment, 2 = it did not affect their adjustment, 3 = it hindered their adjustment).

#### Captivity experiences

At T1 (1991), ex-POWs completed five items tapping various aspects of their captivity experience. *Severity of captivity* was measured via participants' report of weight loss during captivity, which is considered a marker for the hardships of the captivity experience (Sutker, Galina, & West, 1990). In addition, ex-POWs were asked whether they were held in *solitary confinement* during captivity. Three other items evaluated *physical suffering, psychological suffering, and humiliation* in captivity (Tsur, Defrin, Levin, Itzhaky, & Solomon, 2019). These three items were combined into a global *suffering* score, which was created based on a principal component analysis that yielded a single unified factor (factor loadings: physical suffering = 0.89, psychological suffering = 0.95, humiliation = 0.86; see Tsur et al., 2019). Therefore, captivity experience was evaluated by three indices: severity of captivity, solitary confinement, and suffering during captivity.

#### Fear of COVID-19

At T4, participants completed seven items particularly tailored to the COVID-19 experience (Tsur & Abu-Raiya, 2020; Zhen & Zhou, 2020). Participants were asked to rate on a seven-point scale, ranging from 1 (*not at all*) to 7 (*very much*), the extent to which they agreed with each of the items presented to them (“e.g. “I am worried that I will get infected,” “I am afraid that COVID-19 will spread all over the world and will remain for a long time,”). The fear of COVID-19 score was calculated by averaging the responses to all items, with higher scores indicating higher COVID-19 fear. Cronbach's alpha for the current sample was 0.88, indicating high reliability.

#### COVID-19 ASD

At T4, participants completed a 24-item self-report questionnaire, based on the acute stress disorder scale (ASDS; Bryant, Moulds, & Guthrie, 2000), and tailored to the COVID-19 experience. Participants were asked to rate on a five-point scale, ranging from 1 (*not at all*) to 5 (*very much*), how frequent they experienced each of the symptoms since the COVID-19 outbreak. The COVID-19 ASD score was calculated by averaging the responses to all items, with higher scores indicating higher COVID-19 ASD. Cronbach's alpha for the current sample was 0.96, indicating high reliability.

### Data analysis

Little's Missing Completely at Random test (MCAR) revealed that the data were missing completely at random, χ^2^(109) = 114.32, *p* = 0.345 for the whole sample, as well as for the data that included only ex-POWs and contained information about captivity χ^2^(124) = 129.64, *p* = 0.346. Missing data were handled using estimation maximization (EM) while running models in SPSS 25 and including all predictors in the model as auxiliary variables.

First, a series of *t* tests and chi-square analyses were conducted to examine group differences between ex-POWs and controls in demographic and study variables. Second, two hierarchical regression analyses were conducted, examining the unique contribution of war captivity and participants' appraisal of the extent to which their war experiences affected their adjustment to the COVID-19-related lockdown and social restrictions to the prediction of fear of COVID-19 and COVID-19 ASD. In step 1 of these analyses, the effects of participants' age, education, living status, and COVID-19-related occupational status were examined as well as exposure to stressful life events and exposure to COVID-19 at T4. To avoid multicollinearity, number of stressful life events at T2 and T3 were summed to one score. In step 2, we added the specific effects of war captivity (ex-POWs/control group) and participants' appraisal of the impact of their war-related experiences on adjustment to the current quarantine and isolation to the regression model. In this step we also included two interactions: (a) the interaction between war captivity (ex-POWs/control group) and exposure to COVID-19, and (b) the interaction between war captivity (ex-POWs/control group) and the appraisal of the impact of war-related experiences on adjustment to the current quarantine and isolation.

To examine the associations between ex-POWs' captivity experiences as measured at T1 (severity of captivity, being held in solitary confinement, and suffering during captivity) and fear of COVID-19 and COVID-19 ASD, Pearson's correlations and *t* test were conducted. A series of ANOVAs and chi square analyses were conducted to examine the associations between the ex-POWs' appraisal of the impact of war-related experiences on adjustment to the current quarantine and isolation at T4, captivity experiences as measured at T1, and fear of COVID-19 and COVID-19 ASD at T4.

Finally, two hierarchical regression analyses were conducted, with fear of COVID-19 and COVID-19 ASD as the dependent variables. In the first step, ex-POWs' age, education, occupational and living status, exposure to stressful life events at T1, T2, and T3 (number of life events at T2 and T3 were summed to one score), and exposure to COVID-19 at T4, were included as covariates. The second step included the following predictors: Severity of captivity, solitary confinement, and suffering during captivity (as measured at T1) and participants' appraisal of the effect of their captivity experiences on their current adjustment (as measured at T4).

## Results

The two groups did not significantly differ in their exposure to stressful life events nor in their level of exposure to COVID-19 at T4. The two groups did significantly differ, however, in levels of fear of COVID-19 and in COVID-19 ASD symptoms, as ex-POWs reported higher levels of fear of COVID-19 and COVID-19 ASD symptoms than controls (Table 1).

The two groups significantly differed in their appraisal of the extent to which their war-related experiences affected their current adjustment to the COVID-19-related lockdown and social restrictions (see Table 1). Most of the controls (80%) reported that their war-related experience did not affect their adjustment and only 42.5% of the ex-POWs provided this answer. More ex-POWs evaluated their experiences as either facilitating or hindering their current adjustment as compared to controls (see Table 1).

### Predicting fear of COVID-19 and COVID-19 ASD as a function of war captivity

Pearson's correlation yielded a significant moderate association between fear of COVID-19 and COVID-19 ASD, *r* = 0.588, *p* < 0.001. The results of the hierarchical regression models predicting fear of COVID-19 and COVID-19 ASD according to war captivity and participants' appraisal of the effect of their war experiences on their current adjustment are presented in Table 2. The regression model for fear of COVID-19 explained 20.9% of the variance, adjusted *R*^2^ = 17.9%, *F*(11 170) = 4.583, *p* < 0.001. These effects were not biased by multicollinearity (1.04 > VIF > 2.111, 0.49 > tolerance>0.66). As can be seen in Table 2, study group was significantly associated with fear of COVID-19, with ex-POWs reporting higher levels of fear than did controls. In addition, the appraisal of the extent to which war-related experiences affected their adjustment had a unique positive association with fear of COVID-19. Participants' level of education was negatively associated with this fear. Finally, neither exposure to COVID-19 nor the two-way interactions between group and exposure and between group and appraisal made a significant contribution to the prediction of fear of COVID-19.

**Table 2.** Predicting COVID-19 ASD and fear of COVID-19 according to background variables, COVID-19 exposure, appraisal of war experiences and group (*N* = 185)

	Fear of COVID-19					COVID-19 ASD				
	*B*	s.e.	*β*	*t*	*p*	*B*	s.e.	*β*	*t*	*p*
Step 1										
Age (T4)	−0.149	0.106	−0.102	−1.408	0.161	−0.155	0.058	−0.193	−2.690	0.008
Living with spouse (T4)	0.078	0.104	0.053	0.748	0.455	0.069	0.057	0.085	1.217	0.225
Occupational status (T4)	0.168	0.109	0.114	1.538	0.126	0.158	0.059	0.197	2.670	0.008
Education (T4)	−0.425	0.108	−0.290	−3.928	0.000	−0.196	0.059	−0.244	−3.336	0.001
Life events (T1)	0.123	0.107	0.083	1.156	0.249	0.041	0.058	0.051	0.709	0.479
Life events (T3)	−0.038	0.107	−0.025	−0.353	0.724	−0.033	0.058	−0.040	−0.569	0.570
COVID-19 exposure (T4)	0.186	0.107	0.127	1.744	0.083	0.072	0.058	0.089	1.242	0.216
Step 2										
Age (T4)	−0.152	0.104	−0.104	−1.469	0.144	−0.135	0.051	−0.169	−2.662	0.009
Living with spouse (T4)	0.014	0.101	0.009	0.134	0.894	0.016	0.050	0.020	0.319	0.750
Occupational status (T4)	0.027	0.111	0.018	0.244	0.808	0.035	0.054	0.044	0.649	0.517
Education (T4)	−0.394	0.105	−0.269	−3.731	0.000	−0.153	0.052	−0.191	−2.949	0.004
Life events (T1)	0.072	0.103	0.049	0.699	0.485	0.000	0.051	0.001	0.009	0.993
Life events (T3)	−0.049	0.107	−0.032	−0.457	0.648	−0.047	0.052	−0.056	−0.887	0.376
COVID-19 exposure (T4)	0.139	0.104	0.094	1.338	0.183	0.039	0.051	0.049	0.766	0.445
Group	0.293	0.145	0.198	2.024	0.045	0.261	0.071	0.322	3.667	0.000
Appraisal of war experiences (T4)	0.320	0.122	0.216	2.621	0.010	0.276	0.060	0.341	4.612	0.000
Captivity Group *exposure	−0.074	0.138	−0.043	−0.535	0.593	0.068	0.068	0.072	1.012	0.313
Captivity Group*appraisal	0.036	0.077	0.044	0.473	0.637	−0.009	0.038	−0.019	−0.230	0.819

Notes. Group: ex-POWs = 1, controls = 0. Marital status (1 = living with spouse; 0); working status (1 = change due the COIVD-19, 0 = no change). Life events at T3 represent the sum of number of life events at T2 and T3. The appraisal of war experiences refers to the effect of war experiences on adjustment to COVID-19-induced lockdown and home isolation.

The hierarchical regression model for COVID-19 ASD explained 37.8% of the variance, adjusted *R*^2^ = 33.7%, *F* = (11 170) = 9.380, *p* < 0.001. These effects were not biased by multicollinearity (1.019 > VIF > 1.942, 0.515 > tolerance > 0.908). As can be seen in Table 2, study group was significantly associated with COVID-19 ASD, with ex-POWs reporting higher levels of symptoms than controls. In addition, the appraisal of the extent to which war-related experiences affected adjustment to COVID-19-related lockdown and home isolation had a unique positive association with COVID-19 ASD. In addition, age and level of education were negatively associated with COVID-19 ASD. Finally, neither exposure to COVID-19 nor the two-way interactions between group and exposure or between group and appraisal made a significant contribution to the prediction of COVID-19 ASD.

### The contribution of captivity experience to fear of COVID-19 and COVID-19 ASD

Pearson's correlations indicated that severity of captivity measured at T1 was not significantly associated with neither fear of COVID-19 (*r* = −0.13, *p* = 0.169) nor COVID-19 ASD (*r* = 0.014, *p* = 0.880). Suffering during captivity, as measured at T1, was significantly associated with both fear of COVID-19 (*r* = 0.29, *p* = 0.001), and COVID-19 ASD (*r* = 0.26, *p* = 0.004). Being held in solitary confinement during captivity was not significantly related to fear of COVID-19 (*t* = 1.21, *p* = 0.230) nor to COVID-19 ASD (*t* = 0.39, *p* = 0.700).

Ex-POWs' appraisal of the extent to which war-related experiences affected adjustment to COVID-19-induced lockdown and home isolation was not associated with neither severity of captivity nor with suffering during captivity (see Table 3). It was also not associated with being held in solitary confinement during captivity (χ^2^(2) = 1.40, *p* = 0.497). Among those who were held in solitary confinement, 19 (23.5%) reported that their captivity experiences facilitated their adjustment as compared to 5 (13.9%) of those who were not held in solitary confinement; 32 (39.5%) of those who were held in solitary confinement reported it did not affect their adjustment as compared to 16 (44.4%) of those who were not; and 30 (37.0%) of those held in solitary confinement reported their war experiences hindered their current adjustment as compared to 15 (41.7%) of those who were not.

**Table 3.** Captivity experience, fear of COVID-19, and COVID-19 ASD according to appraisal of war experiences among ex-POWs (*N* = 120)

	Facilitate *n* = 24	No effect *n* = 48	Hinder *n* = 45		
	*M* (*s.d.*)	*M* (*s.d.*)	*M* (*s.d.*)	*F* (2, 114)	*p*
Severity of captivity (T1)	11.26 (2.48)	13.96 (5.38)	13.00 (5.22)	2.46	0.090
Suffering during captivity (T1)	3.78 (0.61)	3.64 (0.64)	3.84 (0.82)	0.94	0.394
Fear of COVID-19 (T4)	4.48 (1.39)	4.37 (1.44)	5.45 (1.22)	8.25	<001
COVID-19 ASD (T4)	1.69 (0.51)	1.68 (0.66)	2.06 (0.85)	26.28	<001

Ex-POWs' appraisal of the extent to which war-related experiences affected adjustment to COVID-19-induced lockdown and home isolation was, however, significantly associated with both fear of COVID-19 and COVID-19 ASD. Duncan comparisons indicated that ex-POWs who reported that their war experiences hindered their current adjustment reported higher levels of fear of COVID-19 and COVID-19 ASD as compared to those who reported that they facilitated adjustment or that captivity experiences did not affect their adjustment (see Table 3).

The regression model for fear of COVID-19 explained 33.4% of the variance among ex-POWs, adjusted *R*^2^ = 26.3%, *F*(11 104) = 4.736, *p* < 0.001. These effects were not biased by multicollinearity (0.99 > VIF > 1.86, 0.33 > tolerance > 0.92). As can be seen in Table 4, appraisal of the extent to which war-related experiences affected adjustment to COVID-19-related lockdown and home isolation was significantly associated with fear of COVID-19. In addition, lower level of education and living with spouse were significantly associated with higher levels of fear of COVID-19. Suffering in captivity, as measured at T1, was significantly associated with fear of COVID-19: The higher the level of suffering in captivity, the higher the fear of COVID-19. Interestingly, severity of captivity was negatively associated with fear of COVID-19. Being held in solitary confinement during captivity did not make a significant contribution to the prediction of fear of COVID-19.

**Table 4.** Predicting fear of COVID-19 and COVID-19 ASD according to background variables (*N* = 120)

	Fear of COVID-19					COVID-19 ASD				
	*B*	*s.e.*	*β*	*t*	*p*	*B*	*s.e.*	*β*	*t*	*p*
Step 1										
Age (T4)	−0.055	0.031	−0.157	−1.762	0.081	−0.059	0.018	−0.283	−3.218	0.002
Education (T4)	−0.121	0.034	−0.321	−3.583	0.001	−0.067	0.020	−0.296	−3.341	0.001
Living with Spouse (T4)	1.874	0.610	0.266	3.071	0.003	0.890	0.358	0.213	2.483	0.015
Occupational Status (T4)	0.045	0.113	0.036	0.397	0.692	0.099	0.066	0.134	1.497	0.137
Life events (T1)	0.013	0.025	0.046	0.533	0.595	0.004	0.015	0.021	0.243	0.808
Life events (T3)	−0.129	0.142	−0.079	−0.910	0.365	−0.081	0.083	−0.084	−0.977	0.331
COVID-19 exposure (T4)	0.148	0.088	0.153	1.687	0.094	0.051	0.052	0.089	0.995	0.322
Step 2										
Age (T4)	−0.007	0.033	−0.020	−0.214	0.831	−0.022	0.019	−0.109	−1.208	0.230
Education (T4)	−0.101	0.034	−0.267	−2.947	0.004	−0.035	0.019	−0.156	−1.833	0.070
Living with spouse (T4)	1.277	0.589	0.181	2.167	0.033	0.541	0.329	0.129	1.645	0.103
Occupational Status (T4)	0.010	0.107	0.008	0.097	0.923	0.048	0.060	0.065	0.803	0.424
Life events (T1)	0.010	0.024	0.035	0.408	0.684	−0.008	0.014	−0.048	−0.601	0.549
Life Events (T3)	−0.242	0.137	−0.148	−1.769	0.080	−0.100	0.077	−0.103	−1.311	0.193
COVID-19 exposure (T4)	0.085	0.084	0.087	1.011	0.314	0.009	0.047	0.016	0.198	0.844
Severity of captivity (T1)	−0.073	0.025	−0.249	−2.866	0.005	−0.012	0.014	−0.067	−0.814	0.418
Solitary confinement (T1)	0.039	0.195	0.018	0.198	0.843	−0.178	0.109	−0.139	−1.634	0.105
Suffering during captivity (T1)	0.601	0.191	0.297	3.137	0.002	0.263	0.107	0.219	2.458	0.016
Appraisal of war experiences (T4)	0.429	0.165	0.224	2.608	0.010	0.49	0.092	0.431	5.335	0.000

Solitary confinement: 0 = no, 1 = Yes. Life events at T3 represent the sum of number of life events at T2 and T3. The appraisal of war experiences refers to the effect of war experiences on adjustment to COVID-19-induced lockdown and home isolation.

COVID-19 exposure, and captivity experiences among ex-POWs (*N* = 120).

The regression model for COVID-19 ASD explained 41.1% of the variance among ex-POWs, adjusted *R*^2^ = 34.8%, *F* (11 104) = 6.59, *p* < 0.001. These effects were not biased by multicollinearity (1.02 > VIF > 1.66, 0.33 > tolerance>0.67). As can be seen in Table 4, suffering in captivity, as measured at T1, was significantly associated with COVID-19 ASD at T4: The higher the suffering in captivity, the higher the COVID-19 ASD. The appraisal of the extent to which war-related experiences affected adjustment with the COVID-19-related lockdown and home isolation was also significantly associated with COVID-19 ASD: Ex-POWs who reported that their war experiences hindered their current adjustment reported higher levels of COVID-19 ASD at T4 than other ex-POWs. Solitary confinement and severity of captivity made no significant contribution to ASD. Lower level of education, younger age, and living with spouse were significantly associated with higher levels of COVID-19 ASD in step 1. However, in step 2, the effects of these background variables were not significant.

## Discussion

The findings of the current study indicate that almost five decades after their experience of war captivity, ex-POWs are vulnerable when confronting a new, unfamiliar, and unrelated stressor, such as the COVID-19 pandemic. Although they did not differ from combat veterans who fought in the same war in their level of exposure to COVID-19, they reported higher levels of fear of the COVID-19 as well as higher tendencies to experience intrusive thoughts and dreams of the pandemic, to react with increased arousal when exposed to various cues that remind them the pandemic, and to try to avoid such reminders. Thus, not only the ex-POWs may suffer from higher levels of distress in general (Solomon et al., 1994), their prior experiences made them more susceptible to react to the threat of the pandemic with specific fear and stress reactions. At the same time, the fact that the effects of stressful life events were not statistically significant implies that it is not the effect of prior stress by itself but rather a specific effect of war captivity.

Furthermore, the current findings point out the role of ex-POWs' perceptions of their captivity in predicting their adjustment to the COVID-19 pandemic. That is, ex-POWs' rating of their physical suffering, psychological suffering, and humiliation in captivity, assessed almost 30 years before the COVID-19 pandemic (T1), predicted both fear of COVID-19 and COVID-19-related ASD. This finding emphasize the long-term detrimental effect of the psychological and interpersonal aspects of captivity.

The significance of the subjective perception of captivity in predicting adjustment to the COVID-19 pandemic seem to be even more prominent in light of the lack of detrimental effects of both severity of captivity and being held on solitary confinement during captivity. Previous studies have demonstrated that both severity of the captivity experience, as measured by degree of weight loss, and being held in solitary confinement are associated with long-term PTSD and other manifestations of distress (Hagan et al., 2018; Myers, Kimbrell, Booe, & Freeman, 2005; Neria, Solomon, & Dekel, 2000). Yet, in this study we did not find association between solitary confinement in captivity and fear of COVID-19 and COVID-19 ASD. Severity of captivity, reflected by weight loss in captivity, was not by itself associated with adjustment to COVID-19 pandemic. Yet, when its effect was examined while taking into account other factors in the regression model, severity of captivity was negatively associated with fear of COVID-19. This pattern may be interpreted as a suppression effect (Tzelgov & Henik, 1991). That is, once the shared variance with other variables is controlled for, severity of captivity may somewhat predict improved adjustment to COVID-19 stress.

The findings of this study further emphasize the importance of individuals' appraisal of the effect of war experiences on their adjustment during the pandemic. Firstly, it was shown that ex-POWs and controls differ in their appraisal of this effect. Most of the controls (80%) reported that these experiences had no effect, as compared to 42.5% of the ex-POWs. Indeed, this finding may reflect differences between experiences of combat and captivity. In combat, which may be a traumatic experience in and of itself (APA, 2013), the combatants are often active agents. The experience of captivity, on the other hand, is often characterized by passivity, uncertainty, and helplessness (Walzer, 1969). Thus, while the first experience seems irrelevant to the COVID-19 situation, the second may have a symbolic resemblance to the current circumstances.

Yet it should also be said that there was heterogeneity in ex-POWs' appraisals of the effect of their war experiences on their adjustment during the pandemic. Twenty percent of the ex-POWs reported that these experiences facilitated their adjustment. The salutogenic effect of captivity might be attributed to acquired resources and an elevated sense of efficacy in handling stressful situations, as was suggested by the stress inoculation perspective (Meichenbaum, 2007), or to gaining an appreciation of life, as reflected by the concept of posttraumatic growth (Tedeschi & Calhoun, 2004). This appraisal was not associated with any of the captivity experience indices that were measured (i.e. severity of captivity, suffering during captivity, or being held in solitary confinement). Yet, it was found to be an important indicator for adjustment, as it was associated with both fear of COVID-19 and COVID-19 ASD.

Interestingly, neither the effect of participants' exposure to the COVID-19 nor its interaction with study group (ex-POW/control) on fear of COVID-19 and COVID-19 ASD were statistically significant. These findings indicate that the level of concrete exposure to the threat of COVID-19 was not related to adjustment to COVID-19 among both ex-POWs and combat veterans. These findings are inconsistent with previous studies that document that direct exposure and proximity to the trauma increase the risk for stress reaction (May & Wisco, 2016). This lack of observed effect may be attributed to the limited spread of the pandemic at the time of data collection as is evident by the number of verified cases (0.18% of the Israeli population) as well as by study participants' own reports. In line with the inclusion of indirect exposure to traumatic event as a potential criterion for ASD (APA, 2013), the findings of this study suggest that the threatening effect of the pandemic seems to be related to its symbolic representation rather to concrete exposure.

Our findings revealed that although the same predictors were associated with fear of COVID-19 and COVID-19 ASD, the explained variance of the latter was higher than that of the former. This trend, along with the moderate association between fear of COVID-19 and COVID-19 ASD, implies that these two indices of adjustment are related but do not overlap. This view is in line with the exclusion of intense fear as one of the DSM-5 criterion for ASD (APA, 2013). This exclusion was supported by studies that demonstrate that fear is weakly predictive of stress reactions among trauma survivors and that other posttraumatic emotional reactions (such as anger or shame) also predict these reactions (Bryant, Friedman, Spiegel, Ursano, & Strain, 2011; Friedman, 2013; Pai, Suris, & North, 2017).

The findings of the current study should be considered in light of its limitations. The first is the sample size, especially that of the controls. Another limitation is the absence of an initial assessment, conducted within the first years following the war. Since the first assessment, at which the experience of captivity was measured, was conducted 18 years after the war, it thus may be subject to a recall bias. The lack of assessment of exposure to stressful life events at T4 is another limitation of the study as well as relying on a single item for assessing participants' appraisals of the effect of their war experiences on their adjustment to the COVID-19 pandemic. Finally, the study was conducted among Israeli ex-POWs and combat veterans, all of whom fought in the same war and thus share a similar social and cultural context. Generalizing from these results to other populations, in other periods and other cultures, should be undertaken cautiously.

The findings of the study demonstrate the powerful effect of exposure to traumatic experiences in influencing one's adjustment to an unrelated stress 40 years later. In addition, it demonstrates that not only the experiences themselves, but individuals' perceptions of them, predict subsequent adjustment. Although the COVID-19 pandemic is a global threat, not all individuals experience the same threat. The findings of the current study point to the crucial role of one's past and emphasize the long-term commitment society must make towards those who were exposed to the horrific effects of man-made brutality. These effects may haunt such individuals for many years in a multitude of contexts.
